# Selective serotonin reuptake inhibitors regulate the interrelation between 5-HT and inflammation after myocardial infarction

**DOI:** 10.1186/s12872-023-03378-6

**Published:** 2023-07-08

**Authors:** Lijun Zhang, Nan Lu, Meiyan Liu

**Affiliations:** 1 Department of Psycho-cardiology, Beijing Anzhen Hospital, Capital Medical University, Beijing, 100029 China; 2 Department of Cardiology, the First Affiliated Hospital of Shantou University Medical College, Shantou, Guangdong 515041 China

**Keywords:** 5-HT system, Inflammatory factors, UCMS, Myocardial infarction, Depression, Escitalopram

## Abstract

**Background:**

Acute myocardial infarction (AMI) is a main cause of death all around the world. There is a close relationship between myocardial infarction (MI) and depression. MI patients with untreated depression had higher mortality than those without depression. Therefore, this study aimed to explore the effect of escitalopram in treating a model under MI and unpredictable chronic mild stress (UCMS).

**Methods:**

Male C57BL/6J mice were treated with sham surgery, or MI surgery, or UCMS, or escitalopram (ES) for a consecutive two weeks. And the mice were divided into Sham group, MI group, MI + UCMS group, MI + UCMS + ES group (*n* = 8 in each group). After treatment, the mice went through open field test for anxiety behavior, sucrose preference test for depressive behavior. After sacrificed, the blood, heart, hippocampus, and cortex were collected.

**Results:**

The escitalopram badly increased the area of cardiac fibrosis size. The sucrose preference test demonstrated that escitalopram treatment showed significant effect in improving depressive behaviors of mice under MI + UCMS. The potential mechanism involved the interrelation between 5-HT system and inflammation. MI significantly affected the level of cardiac SERT. Both UCMS and ES significantly affected the level of cortex TNF-α. UCMS significantly affected the level of cardiac IL-33. In the hippocampus tissue, TNF-α was positively correlated with SERT, and IL-10 was positively correlated with SERT. In the cortex tissue, IL-33 was positively correlated with 5-HT_4_R, and sST2 was positively correlated with 5-HT.

**Conclusions:**

Two-week escitalopram treatment might worsen myocardial infarction. But escitalopram could benefit depressive behaviors, which may be related with the interrelationship between the 5-HT system and inflammatory factors in the brain.

## Introduction

Acute myocardial infarction (AMI) is a main cause of death all around the world [1]. There are nearly 610,000 persons suffering from new myocardial infarction each year in the USA [2]. The morbidity and mortality of myocardial infarction remain increasing annually in China, meanwhile, the mortality of AMI is 78.24% in rural area and 60.2% in urban area [3]. There is a close relationship between myocardial infarction (MI) and depression [4, 5], and anxiety [6]. According to the INTERHEART research, the prevalence of depression is 21.7% in patients with AMI. Posttraumatic stress disorder (PTSD) is prevalent among patients who survived from MI, leading to adverse outcomes of patients with MI. It is suggested that the potential mechanism lies in myocardial ischemia induced by psychological stress [7]. What is worse, depression results in poor prognosis for patients post-MI. As reported by the TRIUMPH study (Translational Research Investigating Underlying Disparities in Acute Myocardial Infarction Patients' Health Status), MI patients with untreated depression had higher mortality than those without depression [8]. In a 4.3-year follow-up clinical study, cardiac anxiety after myocardial infarction predicts a higher risk of major adverse cardiac events (MACE) [9].

The pathophysiological mechanisms of depression post-MI involve 5-hydroxytryptamine (5-HT) system [10], 5-HT_2B_ receptor signaling [11], hypothalamic–pituitary–adrenal (HPA) axis [12], inflammation, damage-associated molecular patterns (DAMPs)-associated inflammation [13]. Moreover, the intercorrelation between 5-HT system and inflammation has emerged as a novel mechanism [14].

Selective serotonin uptake inhibitors (SSRIs) are widely used in treating depression, and the effectiveness of SSRIs has been proved successful. Escitalopram, as a kind of SSRIs, has been used in dealing with depression, improving the prognosis of mental stress induced myocardial ischemia (MSIMI) [15]. However, whether it is safe to begin escitalopram treatment after AMI remains confusing. Therefore, we conducted this experiment to explore and the effect of escitalopram in treating a model under AMI and unpredictable chronic mild stress (UCMS) via 5-HT system and inflammation in the brain. It has been proved that escitalopram could improve depressive symptoms within 1–2 weeks [16]. Moreover, the first two weeks are important for treating MI, reducing adverse events and improving cardiac prognosis. According to our former animal experiments, a two-week treatment is effective and economic for AMI mice [17]. Thus we choose a two-week period of treatment.

## Materials and methods

### Animal

**Fig. 1:**
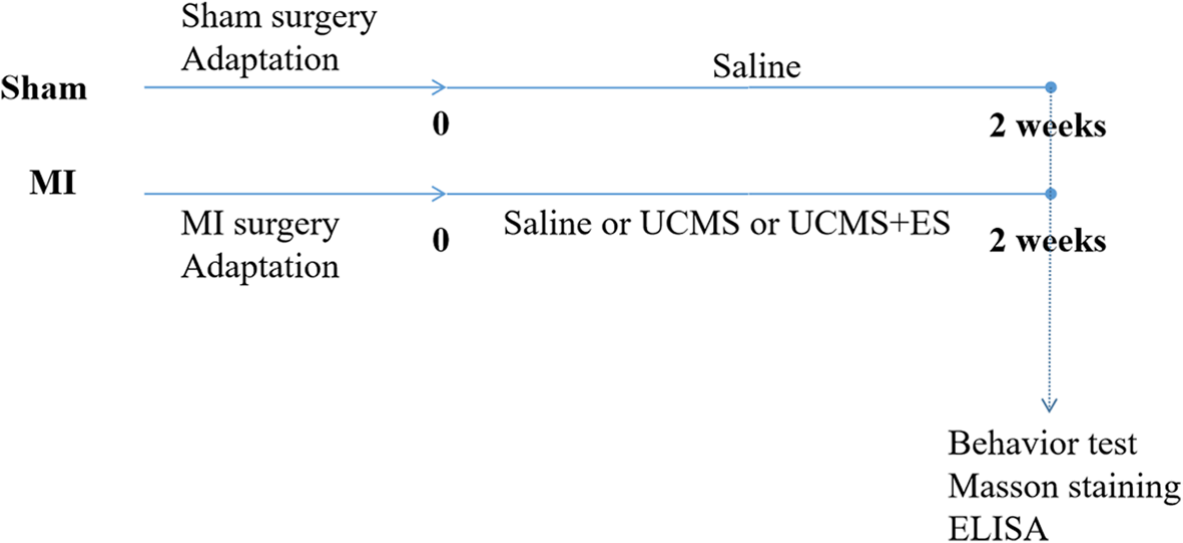
The procedure of the experiment

Eight-week-old male C57BL/6J mice were purchased from SPF (Beijing) Biotechnology Co. Ltd., housing in the Specific Pathogen Free (SPF) experimental animal house (21 °C and 60% humidity). After 5 days of adaptation, 8 mice were given sham surgery, and the successful rate was 100%. 32 mice were given MI surgery, while the success rate was 80%. The mice left were divided into MI group (*n* = 8), MI + UCMS group (*n* = 8), and MI + UCMS + ES group (*n* = 8). UCMS was performed to induce depressive behavior in MI mice [18]. The treatment lasted for 2 weeks. The mice in Sham and MI group received saline treatment. The MI mice in the MI + UCMS group received UCMS and saline treatment, and the MI mice in the MI + UCMS + ES group received UCMS and ES treatment. When the treatment finished, the mice underwent behavior test, including open field test (OFT) and sucrose preference test (SPT). After sacrificing, the blood (*n* = 8, each group), heart, hippocampus and cortex tissues were collected for Masson staining (*n* = 1, each group) or enzyme-linked immunosorbent assay (ELISA) (*n* = 7, each group) (Fig. 1).

### Escitalopram treatment

The mice in the MI + UCMS + ES group were given escitalopram (Denmark Lundbeck) treatment, 4 mg/kg [19] once a day for consecutive 2 weeks. Escitalopram was dissolved in saline, and then administrated to the mice through intragastric gavage. Other mice were given 20 ml/kg saline through intragastric gavage.

### Sham surgery

**Fig. 2:**
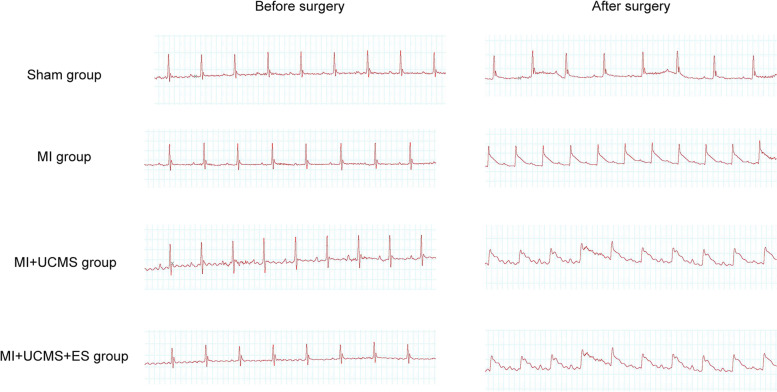
The electrocardiogram before and after sham or MI surgery

After being anesthetized (small animal anesthesia machine, VETEQUIP, VE3525, 2% isoflurane, 1 L/min pure oxygen), the mice were placed on the platform. Then the prethoracic skin was cleared. The electrocardiogram (ECG) monitor was applied for recording ECG variation before and after the surgery. Then the chest was opened and then closed (Fig. 2).

### MI Surgery

The preparations were the same as sham surgery. After opening the chest, the operator exposed the mouse’s heart, and ligated the left anterior descending (LAD) coronary artery fast. Then closed the thoracic cavity as soon as the infarction area turned white. The elevation of ST-segment also revealed the success of the MI surgery (Fig. 2).

### Unpredictable chronic mild stress

The mice in MI + UCMS group and MI + UCMS + ES group underwent UCMS for consecutive two weeks. There were four kinds of UCMS applied in this experiment, such as sound stress for 30 min, no sawdust for 24 h, dampened sawdust for 24 h, tilt cages at 45° against the wall for 30 min [20–22] (Table 1).

**Table 1:** Unpredictable chronic mild stress for 2 weeks

**Day 1**	**Day 2**	**Day 3**	**Day 4**	**Day 5**	**Day 6**	**Day 7**
No sawdust for 24 h	Dampened sawdust for 24 h	Sound stress for 30 min	Tilted cage for 30 min	No sawdust for 24 h	Dampened sawdust for 24 h	Tilted cage for 30 min
**Day 8**	**Day 9**	**Day 10**	**Day 11**	**Day 12**	**Day 13**	**Day 14**
Sound stress for 30 min	Dampened sawdust for 24 h	Tilted cage for 30 min	No sawdust for 24 h	Sound stress for 30 min	Dampened sawdust for 24 h	Tilted cage for 30 min

### Open field test

OFT is a standard protocol to estimate the anxiety behaviors of mice [23, 24]. Each mouse went through the OFT for 6 min, the first 2 min for adaptation, and the next 4 min for recording. Meanwhile, the freezing time, the number of crossing grid, stand-up times, the time spent in the center region, the total moving distances, total distance in the center region, and average velocity were recorded. (Cleversys TopScanlite, USA).

### Sucrose preference test

SPT was used to evaluate the depressive behaviors of mice [25, 26]. The test lasted 48 h. In the first 24 h, the mice were kept alone and adapted sucrose water. In the next 24 h, the weights of sucrose water bottle and pure water bottle were recorded. The sucrose preference calculation formula was as follows: (sucrose consumption/total water consumption) × 100%.

### Masson staining

Masson staining was used for cardiac fibrosis semiquantitative assessment. There were several steps in the Masson staining: hearts fixation, paraffin wax embedding, slices cutting, Masson staining. Then the image was scanned by a panoramic scanner (Pannoramic DESK/MIDI/250/1000), and the area of cardiac fibrosis was analyzed by Image-Pro Plus 6.0 (Media Cybemetics, U.S.A).

### Enzyme-linked immunosorbent assay

The plasma, cardiac, hippocampus, cortex tissues were collected for ELISA. ELISA was conducted to detect the levels of 5-HT (INSJ203223M, Inselisa, Hubei, China), 5-Hydroxytryptamine type 4 receptor (5-HT_4_R, INS201090M, Inselisa, Hubei, China), serotonin transporter (SERT, INS200394M, Inselisa, Hubei, China), tumor necrosis factor (TNF)-alpha (TNF-α, INS202412M, Inselisa, Hubei, China), Interleukin (IL)-10 (IL-10, INS203075M, Inselisa, Hubei, China), IL-33 (INS203053M, Inselisa, Hubei, China), soluble tumour suppressor-2 (sST2, MM-45321M1, Jiangsu Meimian Industrial Co., Ltd, Jiangsu, China), tumor necrosis factor receptor 1 (TNFR1, MM-45934M1, Jiangsu Meimian Industrial Co., Ltd, Jiangsu, China), TNFR2 (MM-46101M1, Jiangsu Meimian Industrial Co., Ltd, Jiangsu, China). ELISA detection was performed according to the instruments as described in the ELISA kits.

### Statistical analysis

One-Sample Kolmogorov–Smirnov Test was used for normal distribution. Three-way (MI × UCMS × drug treatment) analysis of variance (ANOVA) was applied for data analysis. The correlations between 5-HT system and inflammatory factors were analyzed by linear correlation. Pearson correlation test was used for normal distribution data, and Spearman correlation test was used for non-normal distribution data. Then linear regression was tested when there was significant correlation. *P* < 0.05 was considered to be significant different.

## Results

### The myocardial infarction size

**Fig. 3:**
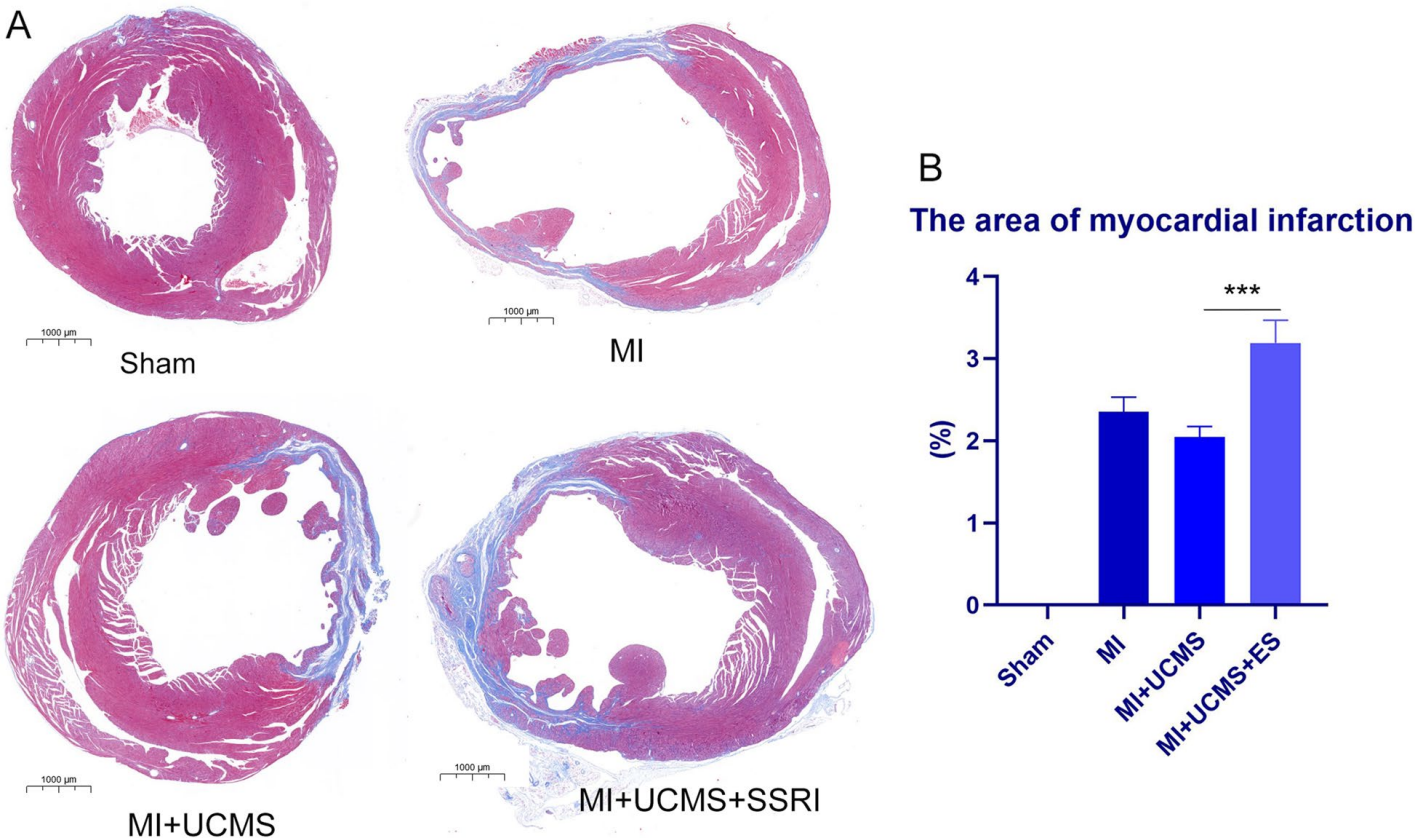
**A** The Masson staining of the ventricle in different groups, the scale bar was show on the Fig. 1000um. **B** The myocardial infarction area comparisons

The Masson staining presented the cross section of the ventricle. The MI surgery enlarged the left ventricle and made the myocardium thinner in MI, MI + UCMS, and MI + UCMS + ES groups. The UCMS did not seem to worsen the enlarged ventricle. The blue color indicated cardiac fibrosis after MI (Fig. 3A). The semiquantitative assessment showed that MI (F_1,11_ = 270.964, *P* = 0.000), ES (F_1,11_ = 63.829, *P* = 0.000) badly increased the area of cardiac fibrosis size. While UCMS had no significant effect of myocardial infarction size (F_1,11_ = 4.567, *P* = 0.065) (Fig. 3B).

### Behavior tests

#### Anxiety behaviors after escitalopram treatment

The open field test which was used to evaluate the anxiety behaviors indicated that MI (F_1,30_ = 0.896, *P* = 0.352), UCMS (F_1,30_ = 0.022, *P* = 0.883), ES (F_1,30_ = 1.681, *P* = 0.206) had no significant effect on freezing time. MI (F = 1.520, *P* = 0.228), UCMS (F = 0.146, *P* = 0.705), ES (F = 0.244, *P* = 0.626) had no significant effect on the number of crossing grid. MI (F = 0.001, *P* = 0.978), UCMS (F = 0.223, *P* = 0.641), ES (F = 0.164, *P* = 0.689) had no significant effect on the stand-up times. MI (F_1,30_ = 0.871, *P* = 0.359), UCMS (F_1,30_ = 0.436, *P* = 0.515), ES (F_1,30_ = 0.180, *P* = 0.674) had no significant effect on the time spent in the center region. MI (F = 0.367, *P* = 0.550), UCMS (F = 0.476, *P* = 0.496), ES (F = 0.044, *P* = 0.835) had no significant effect on the total distance in the center region. MI (F_1,30_ = 1.745, *P* = 0.198), UCMS (F_1,30_ = 0.200, *P* = 0.658), ES (F_1,30_ = 0.099, *P* = 0.755) had no significant effect on total moving distances. MI (F = 1.491, *P* = 0.233), UCMS (F = 0.000, *P* = 0.999), ES (F = 0.687, *P* = 0.414) had no significant effect on the average velocity (Table 2).

**Table 2:** The results of the open field test

	**MI**		**UCMS**		**ES**	
	F_1,30_	*P*	F_1,30_	*P*	F_1,30_	*P*
Freezing time	0.896	0.352	0.022	0.883	1.681	0.206
The number of crossing grid	1.520	0.228	0.146	0.705	0.244	0.626
Stand-up times	0.001	0.978	0.223	0.641	0.164	0.689
Time spent in the center region	0.871	0.359	0.436	0.515	0.180	0.674
Total distance in the center region	0.367	0.550	0.476	0.496	0.044	0.835
Total moving distances	1.745	0.198	0.200	0.658	0.099	0.755
average velocity	1.491	0.233	0.000	0.999	0.687	0.414

#### Depressive behaviors after escitalopram treatment

**Fig. 4:**
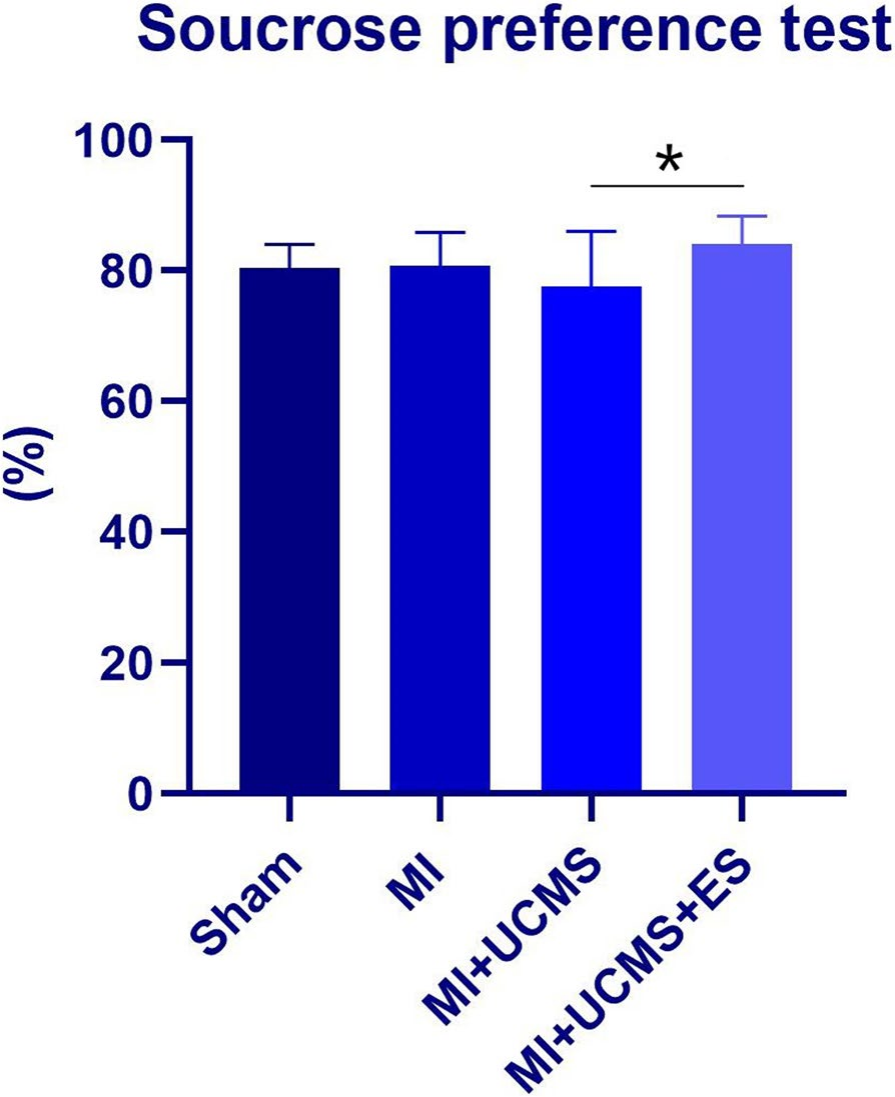
The results of sucrose preference test

The lower sucrose preference demonstrated that the mice expressed depressive behaviors. MI (F_1,30_ = 0.010, *P* = 0.919) and UCMS (F_1,30_ = 1.219, *P* = 0.279) had no significant effect on sucrose preference, while escitalopram treatment (F_1,30_ = 5.106, *P* = 0.032) showed significant effect. Escitalopram treatment effectively improved the depressive behaviors of mice under MI + UCMS (Fig. 4).

### The 5-HT system

**Fig. 5:**
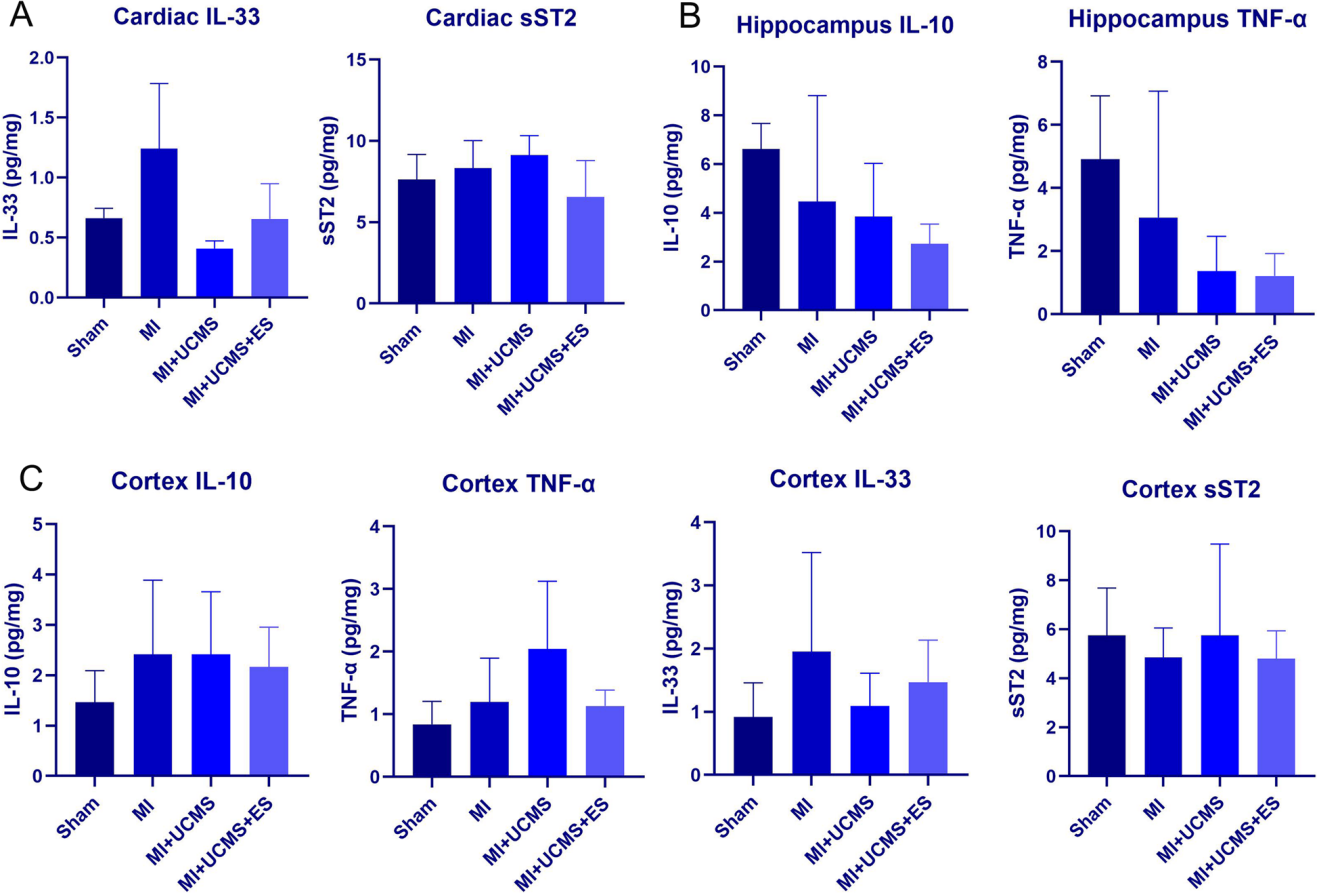
**A** The level of 5-HT, 5-HT_4_R, and SERT in cardiac tissue; **B **The level of 5-HT, 5-HT_4_R, and SERT in hippocampus tissue; **C **The level of 5-HT, 5-HT_4_R, and SERT in cortex tissue

MI (F_1,29_ = 0.317, *P* = 0.578), UCMS (F_1,29_ = 0.319, *P* = 0.577), ES (F_1,29_ = 0.496, *P* = 0.488) had no effect on plasma 5-HT. MI significantly affected the level of cardiac SERT (F_1,11_ = 9.730, *P* = 0.014), while UCMS (F_1,11_ = 2.521, *P* = 0.151) and ES (F_1,11_ = 0.407, *P* = 0.541) had no significant effect on cardiac SERT. MI, UCMS, ES had no effect on cardiac 5-HT, 5-HT_4_R. MI, UCMS, ES had no effect on 5-HT, SERT, 5-HT_4_R of hippocampus and cortex (Fig. 5, Table 3).

**Table 3:** 5-HT system and inflammatory factors

	**Myocardium**			**Hippocampus**			**Cortex**		
	MI	UCMS	ES	MI	UCMS	ES	MI	UCMS	ES
**5-HT**	0.285	3.357	1.740	0.017	3.150	0.113	0.728	2.015	0.089
**5-HT**_**4**_**R**	0.304	0.012	0.290	0.005	0.219	0.003	2.738	3.757	0.019
**SERT**	9.730^*^	2.521	0.407	0.118	1.719	0.001	0.357	2.667	0.851
**IL-10**	0.000	0.068	1.736	1.089	0.088	0.298	2.299	0.000	0.188
**IL-33**	5.122	10.257^*^	0.93	-	-	-	3.930	2.946	0.607
**sST2**	0.254	0.323	3.378	-	-	-	0.458	0.490	0.588
**TNF-α**	-	-	-	0.936	0.796	0.006	0.788	4.811^*^	6.044^*^
**TNFR1**	-	-	-	-	-	-	0.174	0.178	0.018
**TNFR2**	-	-	-	-	-	-	0.015	0.217	0.008

^*^P < 0.05, ^**^P < 0.01, ^***^P < 0.001

### The inflammatory factors

**Fig. 6:**
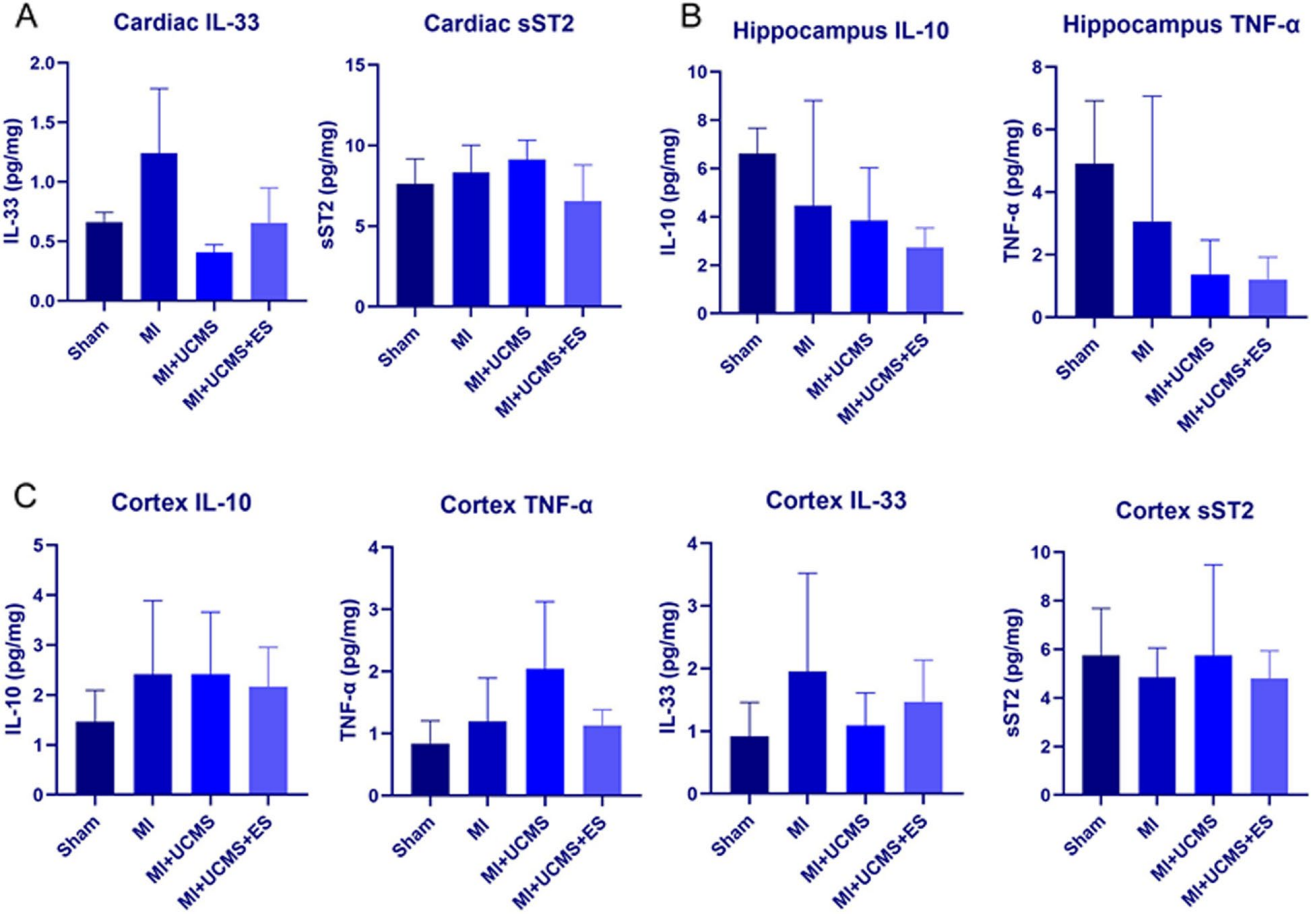
**A **The level of IL-33 and sST2 in cardiac tissue; **B** The level of IL-10 and TNF-α in hippocampus tissue; **C **The level of IL-33, sST2, IL-10 and TNF-α in cortex tissue

The ELISA detection showed that UCMS (F_1,11_ = 10.257, *P* = 0.011) significantly affect the level of cardiac IL-33, which level was lower in UCMS than sham and MI, while MI (F_1,11_ = 5.122, *P* = 0.50) and ES (F_1,11_ = 0.93, *P* = 0.363) had no effect on cardiac IL-33 (Fig. 6A). Both UCMS (F_1,25_ = 4.811, *P* = 0.005) and ES (F_1,25_ = 6.044, *P* = 0.021) significantly affect the level of cortex TNF-α, which level was higher in UCMS but lower in ES than sham. MI, UCMS, ES had no effect on other inflammatory factors of the myocardium, hippocampus, and cortex (Fig. 6B-C, Table 3).

### The correlation between 5-HT system and inflammatory factors in the brain

**Fig. 7:**
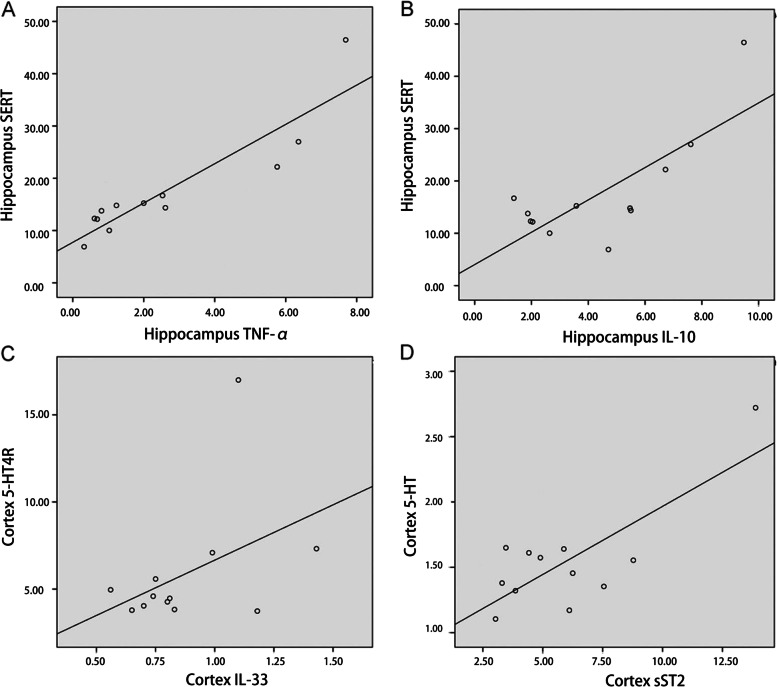
**A** The linear correlation of hippocampus TNF-α and SERT; **B** The linear correlation of hippocampus IL-10 and SERT; **C** The linear correlation of cortex IL-33 and 5-HT_4_R; **D** The linear correlation of cortex sST2 and 5-HT

In the hippocampus tissue, TNF-α was positively correlated with SERT (r = 0.909, *P* = 0.000), and IL-10 was positively correlated with SERT (r = 0.767, *P* = 0.004). Moreover, the unary linear regression analysis also showed a positive correlation between TNF-α and SERT (*R*^2^ = 0.826, *P* = 0.000), IL-10 and SERT (*R*^2^ = 0.589, *P* = 0.004) (Fig. 7A-B, Table 4).

**Table 4:** The correlation between 5-HT system and inflammatory factors

	**Myocardium**			**Hippocampus**			**Cortex**		
	**5-HT**	**5-HT**_**4**_**R**	**SERT**	**5-HT**	**5-HT**_**4**_**R**	**SERT**	**5-HT**	**5-HT**_**4**_**R**	**SERT**
**TNF-α**	-	-	-	0.128	-0.189	0.909^***^	0.128	-0.189	-0.344
**IL-10**	0.135	0.532	-0.068	0.366	-0.120	0.767^**^	0.366	-0.120	-0.262
**IL-33**	-0.235	-0.428	-0.459	-	-	-	-0.092	0.415^*^	0.347
**sST2**	0.505	0.336	0.178	-	-	-	0.741^***^	0.058	-0.002
**TNFR1**	-	-	-	-	-	-	0.076	-0.148	0.057
**TNFR2**	-	-	-	**-**	**-**	**-**	0.169	-0.098	-0.091

^*^P < 0.05, ^**^P < 0.01, ^***^P < 0.001

In the cortex tissue, IL-33 was positively correlated with 5-HT_4_R (*r* = 0.415, *P* = 0.039), and SST2 was positively correlated with 5-HT (*r* = 0.741, *P* < 0.001). Moreover, the unary linear regression analysis also showed a positive correlation between IL-33 and 5-HT_4_R (*R*^2^ = 0.172, *P* = 0.039), sST2 and 5-HT (*R*^2^ = 0.548, *P* < 0.001) (Fig. 7C-D, Table 4).

## Discussions

The study presented that there was a significant interrelation between the 5-HT system and inflammatory factors in the brain of mice under MI or UCMS. Moreover, the escitalopram treatment could benefit depressive behavior, but could not reduce the myocardial infarction size, which may be related with the 5-HT system and inflammation in the brain.

In this experiment, UCMS was applied while giving escitalopram to the mice in the MI + UCMS + ES group at the same time, without determining whether the animals were depressed or not. We would like to address this methodology here: (1) It is normal and standard method of animal experiment, which has been used widely in many published papers [27–29] and our own studies [17, 30] (2) in order to reflect the real clinical practical situation as much as possible, we chose this method. In clinical practice, patients are always suffering from psycho-social distress while receiving antidepressant treatment. (3) The animals that had just undergone surgeries, are too weak to go through behavior tests. It is not accurate to evaluate their depressive or anxiety behaviors under such situation. Therefore, it is better to estimate their behaviors after 2-week treatment.

5-HT is a kind of neurotransmitter in the brain, and plays a crucial role in maintaining mental health. The reduction of 5-HT in the synaptic cleft leads to depression [31]. In addition, 5-HT is regarded as a vasodilation and vasoconstriction factor depending on the intact endothelium [32]. 5-HT takes place as a vasodilation factor when the vascular endothelium is in good condition, on the contrary, it constricts the blood vessels while the vascular endothelium is injured [33]. During the process of acute myocardial infarction, 5-HT binds to the 5-HT_2A_ receptor of platelets, inducing platelet aggregation, thrombogenesis, vasoconstriction and aggravating cardiac damage [34]. However, escitalopram produced no effect on serotonergic system in the experiment. We suggest the potential reasons involved: (1) The results are affected by the treatment period and dose of escitalopram [16]. (2) The concentration of 5-HT is influenced by the biosynthesis and metabolism of 5-HT [35]. Therefore, it would be better to detect the rate-limiting enzyme (such as tryptophan hydroxylase) and metabolism production (5-hydroxyindole acetic acid, 5-HIAA), which could indirectly reflect the concentration of 5-HT. (3) 5-HT is produced in the enterochromaffin and raphe nucle, moreover, 5-HT receptor and SERT are widely distributed in the central nerves system [36], cardiovascular system [37], gastrointestinal system [38] and platelets [39]. Two-week treatment of escitalopram may exert an effect on other systems. Due to the limited detection methods in this experiment, we are unable to detect the 5-HT in synaptic cleft, or 5-HT system in other parts of the body. (4) Inflammation disturbs the effect of escitalopram. Furthermore, 5-HT has also been recognized as pro-inflammatory factor in the peripheral immune system, contributing to immune regulation [40]. However, inflammatory cytokines interfere the synthesis of 5-HT, leading to the reduction of 5-HT [14].

AMI patients suffer a high prevalence of depression. SSRIs has been regarded as one kind of first-line medication of treating depression [41]. The safety of taking antidepressant medicine for patients with cardiovascular diseases has already been discussed [42]. Actually, the question has been raised for a long time, that whether antidepressant treatment should be considered in patients short after acute coronary syndrome. Several researches had been conducted to solve the question, while inverse outcomes appeared due to the different follow-up periods and sample size. Nina Rieckmann et al. presented the worse effect of SSRIs for patients with acute coronary syndrome (ACS) [43]. On the other hand, after 8-year follow-up, a randomized clinical trial has proved the safety and better cardiac prognosis of escitalopram, whilst treating patients shortly after ACS for a consecutive 24 weeks [44]. In our study, the two-week escitalopram treatment does not benefit myocardial infarction, indicating that AMI patients should be monitored carefully, during the first 2-week escitalopram treatment.

Following myocardial infarction, severe inflammation response occurs in the cardiac tissue, contributing to cardiac repair, fibroblasts proliferate, and infarct myofibroblasts [45]. Our study chose sever representative inflammatory factors such as IL-33, TNF-α, IL-10, and the receptors of TNF-α and IL-33. Both sST2 and ST2L are the receptors of IL-33, while the two receptors exert different functions. The IL-33/ST2L signaling pathway plays a vital role in heart protection, while sST2 inhibits the signaling pathway and contributes to heart injury [46]. Our study found that UCMS lowered the level of cardiac IL-33, suggesting that UCMS attenuated the protection of IL-33 to the heart. IL-33 upregulates the level of sST2, which indicates the degree of cardiac injury and the adverse cardiac outcomes [47]. During the two-week treatment of escitalopram, our study presented the anti-depressant effect of escitalopram in mice under MI + UCMS, but not anti-anxiety effect. It has been widely acknowledged that the mechanism of escitalopram in treating depression is inhibiting SERT from reuptaking 5-HT in the synaptic cleft [48]. While Yu Sun et al. [49] found that the kynurenine pathway metabolite levels were negatively associated with the anti-depressant effect of escitalopram. In addition, recent researchers carry out that escitalopram is related with reducing the concentration of prefrontal cortex (PFC) and hippocampus inflammatory cytokines [50], our study shows that escitalopram reduces the level of TNF-α in cortex and attenuates the inflammatory response. It has been proved that inflammation could be related with 5-HT metabolism [51]. In our previous experimental study, we have revealed the positive association between IL-1β and 5-HT in hippocampus, and negative association between IL-1β and 5-HT in median raphe nucleus and cortex [52]. Moreover, this study further explores the relationship between 5-HT system and inflammatory factors, indicating that TNF-α was positively correlated with SERT, and IL-10 was positively correlated with SERT in hippocampus, IL-33 was positively correlated with 5-HT_4_R, and sST2 was positively correlated with 5-HT in cortex. In the astrocytes experiment, the upregulation of TNF-α contributes to the rise of SERT via p38 MAPK activation, which enhancing the reuptaking of 5-HT [53]. Therefore, the elevation of TNF-α in brain is associated with depression. In the intestinal epithelial cells experiment, the SERT activation depends on the concentrations of IL-10. The higher level of IL-10 activates SERT, while the lower level inhibits SERT [54]. While no published papers have been found to elucidate the relationship between IL-33 and 5-HT_4_R, sST2 and 5-HT in cortex. Further research needs to be conducted on this subject.

### Limitations

Due to the shortness of research time and funding, there are some limitations in this study: (1) The escitalopram treatment only lasted two weeks, thus, we only explored the initial effectiveness of escitalopram treatment, which was deficient in studying different stages of the whole therapeutic process. (2) We didn’t investigate the potential signaling pathway of the interrelationship between 5-HT system and inflammatory factors. (3) We didn’t design a cell experiment to validate the vivo findings. Therefore, we plan to make up the limitations in our further study.

## Conclusions

Two-week escitalopram treatment might worsen myocardial infarction, therefore, AMI patients should be monitored carefully, during the first 2-week escitalopram treatment. But escitalopram could benefit depressive behaviors, which may be related with the interrelationship between the 5-HT system and inflammatory factors in the brain.

## Data Availability

The datasets used and/or analyzed during the current study available from the corresponding author on reasonable request.
